# Potential of Nitric Oxide and Bacteriophages as Combined Antibacterial Agents to Counter Drug-Resistant Infections

**DOI:** 10.1021/acsabm.5c01489

**Published:** 2025-11-27

**Authors:** Sarah N. Wilson, Vijay Singh Gondil, Elizabeth J. Brisbois, Hitesh Handa

**Affiliations:** † School of Chemical, Materials, and Biological Engineering, 1355 University of Georgia, Athens, Georgia 30605, United States; ‡ Department of Pharmaceutical & Biomedical Sciences, 1355 University of Georgia, Athens, Georgia 30605, United States

**Keywords:** nitric oxide, bacteriophage, antimicrobial resistance, biomedical, combination therapy

## Abstract

The ongoing threat of antimicrobial-resistant (AMR) bacteria and the growing population of AMR bacteria have inspired research into alternative antimicrobial agents. Previous studies have shown clinically relevant bactericidal effects of the molecule nitric oxide (NO). Not only has extensive research proven its antimicrobial effect, but bacteria have also been shown to be less likely to become resistant to NO. Numerous studies have also demonstrated that NO is compatible with commercially available antibiotic drugs, enhancing antimicrobial effects. However, these drugs are not always readily available or easily manufactured. This study proposes combining NO with naturally sourced antibacterial agents, namely bacteriophages. This combination of NO with bacteriophages in solution demonstrated an 82 ± 1.7% killing efficiency against its target pathogen, *Escherichia coli*, and a 74 ± 2.9% reduction in Methicillin-resistant *Staphylococcus aureus* throughout a 12 h growth curve, indicating significant potential for further development as a broad-spectrum antimicrobial combination therapy.

## Graphical abstract

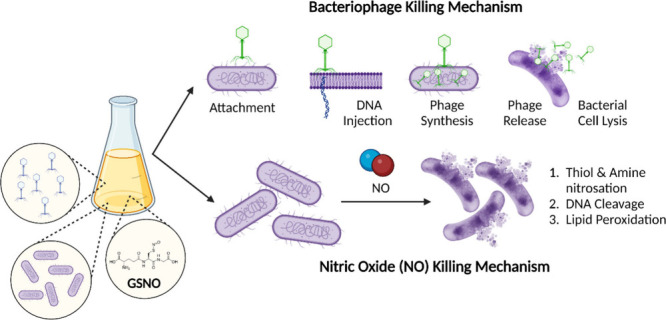

## 1 Introduction

The Infectious Disease Society of America has made numerous statements since the early 2000s regarding the threat of antimicrobial resistance (AMR) and declared the world to be in an emerging crisis of AMR in 2008.^1^ An update from the Centers for Disease Control & Prevention (CDC) in 2019 estimated that 2.9 million antibiotic-resistant infections and 35,000 related deaths occur in the United States annually.^2^ As the rate of AMR increases, the number of newly approved antimicrobial drugs ready for marketing has decreased in the US.^3^ It has become increasingly common to treat infections that were once easily treatable with multiple antibiotics to combat advanced infections. However, these methods increase the likelihood of the pathogens growing resistant to various drug species, ultimately adding to the level of AMR.^1^ The increasing emergence of multidrug-resistant bacteria has prompted research into alternative therapies beyond conventional antibiotics.

Potential alternatives to commonly used antibiotics are molecules that induce stress, membrane lysis, and DNA cleavage in bacteria, such as nitric oxide (NO). Nitric oxide is an endogenous free radical molecule with multifaceted roles in regulatory pathways, inducing harmful consequences, and acting as a protective antioxidant.^4^ It plays a vital role within the immune system, inducing oxidative and nitrosative stress on invading pathogens when released at high concentrations (>1 μM).^5^ Additionally, due to its nonspecific antimicrobial properties, bacteria cannot develop resistance to NO as quickly as commercialized antimicrobial drugs.^6,7^ A recent study by Rouillard et al. developed biopolymers that released NO to kill bacteria in conjunction with conventional antibiotics used to treat similar infections; they found that the presence of NO increased bacterial susceptibility to the antibiotics.^8^ Similar results were produced when combining NO with antifungal drugs and antimicrobial peptides.^9–11^ The continued inclusion of NO into biocompatible medical devices has the potential to significantly decrease the risk of developing an AMR infection.

Another alternative to conventional antimicrobials is the use of bacteriophages (phages), natural parasites of bacteria. The study of phages has been increasing in popularity consistently since their description and discovery in 1915 and 1917 by Frederick Twort and Félix d’Hérelle, respectively.^12^ Phages are naturally sourced, highly specific, and cost-effective, which all lend to the attractiveness of using them as antimicrobial agents compared to developing new expensive antibiotic molecules.^13^ As their popularity increased, more benefits of using phages as antibacterial agents have arisen, such as their specificity toward a single pathogen, not to damage commensal microbiota, a lack of side-effects after phage administration, and last, they are self-regulating entities and will only decrease in number once the targeted pathogen has cleared as well.^1,13,14^ Similar to NO, studies have shown that phage resistance develops exponentially slower than AMR due to the phages’ ability to coevolve rapidly with their host cells.^15^

The exploration of NO+phage combination therapies has rarely been studied. Previous studies have shown NO’s broad-spectrum killing capacity and phage’s adaptability and biocompatibility, but also outlined NO’s short half-life and toxicity along with phage’s high specificity. This study aims to provide an *in vivo*-validated system demonstrating the intentional synchronization of NO release and phage lytic infection to lengthen NO’s therapeutic lifetime and increase bacteriophage’s killing capabilities. Herein, the use of NO and phages in a combined therapy to kill opportunistic pathogens in solution is explored as a proof-of-concept. The water-soluble NO donor, *S*-nitrosoglutathione (GSNO), was used in this study because it resembles naturally produced donors, unlike other commonly studied NO donors. *E. coli* phages (ECΦ) and GSNO were introduced separately and in a combined manner to *E. coli* before growth curve analyses were taken. Additional studies, including double-layer agar plaque assays (DLPAs) and NO release characterizations, were conducted to ensure that the two therapeutic agents did not interfere with each other when in solution. Finally, this novel antibacterial combination was tested against common AMR pathogens to determine its synergistic bactericidal effects.

## 2 Materials and Methods

### 2.1 Materials

Bacteriophages were harvested from the North Oconee Water Reclamation facility in Athens, GA. The bacterial strains *Escherichia coli* (ATCC 25922) and Methicillin-resistant *Staphylococcus aureus* (ATCC BAA41) were obtained through the American Type Culture Collection (Manassas, VA). Lysogeny broth (LB) and agar were purchased from Sigma-Aldrich (St. Louis, MO) along with all components of phage buffer – Tris base, NaCl, MgCl_2_, and CaCl_2_. Deionized water (DI) (18.2 MΩ) was prepared in-house using a distillation unit from Mettler Toledo (Columbus, OH). Gas tanks needed for NOA (nitrogen and oxygen) were purchased from Airgas (Kennesaw, GA). All bacterial experiments were completed in a biosafety level 2 laboratory approved by the University of Georgia.

### 2.2 Methods

#### 2.2.1 GSNO Synthesis

*S*-nitrosoglutathione was synthesized as previously reported.^16^ In short, reduced glutathione was dissolved in 2 M HCL and DI water and chilled over an ice bath. Excess sodium nitrite was added, and the reaction was allowed to proceed for 60 min. The GSNO was precipitated using chilled acetone and collected via vacuum filtration. The GSNO was washed and dried under vacuum for ∼4 h; it was then further dried overnight in a desiccator before testing its purity via chemiluminescent NO release. No GSNO below a purity of 90% was used throughout this study; pure GSNO was stored at −20 °C protected from light.

#### 2.2.2 Harvesting and Purifying of ECΦ

Phages were harvested from sewage water samples at the North Oconee Water Reclamation Facility, Athens, GA, USA. Samples were collected using 50 mL sterile Falcon tubes and transported on ice. Any sediment in the samples was centrifuged at 4500 rpm for 10 min, and the supernatant was removed and filtered through a 0.2 μm sterile syringe filter. The filtered supernatant was then stored at 4 °C before further processing.

For this study, *E. coli* ATCC 25922 (Manassas, VA 20108) was used as the host bacterium for isolating phages from filtered water samples. Overnight cultures were inoculated in lysogeny broth (LB) and grown at 37 °C while shaking at 150 rpm. Once the cultures reached the logarithmic growth stage, as visualized by turbidity, the water samples were added in a 1:1 ratio with phage buffer, comprised of Tris, NaCl, MgCl_2_, and CaCl_2_, at pH 7.5. This mixture was incubated for 2–3 days at 37 °C with shaking at 150 rpm. The phages were isolated through centrifugation at 12,000 rpm for 10 min, after which the supernatant was collected and filtered using a 0.2 μm syringe filter. The resulting supernatant was then combined with a freshly grown *E. coli* culture at a 1:3 ratio. The mixture was combined with 5 mL of 0.7% soft agar and plated atop LB agar plates before incubation at 37 °C overnight. To further grow the phage stock, a single plaque observed on these plates was picked off and resuspended in phage buffer, then centrifuged once more at 10,000 rpm for 5 min. The resulting phage supernatant was syringe-filtered and incubated with the *E. coli* host before being plated as detailed above. This purification procedure was repeated once more to obtain a pure bacteriophage stock solution (ECΦ). Prior to the completion of this study, the phages were characterized through Transmission Electron Microscopy (TEM) to show individual phages floating in solution.^17^ The phages have similar morphology to the bacteriophage family *Podoviridae* based on the International Committee on the Taxonomy of Viruses (ICTV).^18^

#### 2.2.3 Determination of ECΦ Inhibition Due to GSNO

Before analyzing the individual and combined antibacterial efficacy of GSNO and ECΦ at varying concentrations, an examination was conducted of how the individual components affect each other’s efficacy. To determine how different concentrations of GSNO affect the phages’ ability to inhibit bacterial growth, the two were incubated together at varying concentrations of GSNO (10 mM and 5 mM). Purified bacteriophages were serially diluted in LB media until reaching an approximate concentration of 10^7^ PFU mL^–1^. Simultaneously, GSNO was dissolved in LB broth at 40 mM. The GSNO solution and ECΦ suspensions were combined to create solutions containing 10 mM GSNO and 10^6^ PFU mL^−1^; this process was performed in triplicate. Once combined, the solutions were serially diluted and plated within 5 mL of a soft agar at 0.7% with *E. coli* cultures and the GSNO + ECΦ dilutions. Each dilution was plated atop LB agar plates and incubated at 37 °C overnight before analysis. This procedure was repeated for 5 mM GSNO and plated at 2 time points per concentration (0 and 12 h).

#### 2.2.4 Analyzing NO Release Variations with the Inclusion of ECΦ

Nitric oxide release was analyzed via a Sievers Nitric Oxide Analyzer (NOA) model 280i (Boulder, CO), which utilizes chemiluminescence-based detection. Briefly, suspensions of 10^7^ PFU mL^–1^ + 100 or 50 mM GSNO were made up in LB broth. Each solution was diluted into an amber collection vial containing LB broth and 1% antifoam to obtain final concentrations of 10^6^ PFU mL^–1^ + 10 or 5 mM GSNO. During analysis, the treatments were kept at 37 °C by a circulating water bath to mimic physiological conditions. Nitric oxide release was calculated as a steady purge stream of nitrogen gas with a flow rate of ∼200 cm^3^ min^–1^, which was swept into the LB broth containing each sample solution. The inert gas then carried the emitted nitric oxide from the sample solutions into the reaction chamber, where the following reactions take place.

$$NO+O_{3}\to {NO}_{2}^{*}+O_{2}$$

$${NO}_{2}^{*}\to NO_{2}+hv$$

The emitted photon is detected within the NOA by a photomultiplier tube and is output as a measurement of NO in parts per billion (ppb). An NOA constant (mol ppb^–1^ sec^–1^) was then used to determine the moles of NO released per minute. Each sample was read in triplicate and GSNO solutions without phages were used as controls.

#### 2.2.5 Antibacterial Evaluation of GSNO and ECΦ

##### 2.2.5a Individual Quantifications

Bactericidal efficacy was analyzed for each ECΦ and GSNO separately before combining efforts. An *E. coli* culture was grown in LB broth until the optical density at 600 nm (OD_600_) reached 0.1, corresponding to approximately 10^8^ CFU mL^–1^. Equal volumes of bacterial culture were added to every well within a 96-well plate. Simultaneously, ECΦ dilutions were made using LB media starting from a stock of 2 × 10^10^ PFU mL^–1^; ECΦ dilutions ranged from 10^3^ – 10^9^ PFU mL^–1^ and were added to the previously plated *E. coli* cultures at half the volume (Table 1). Separately, GSNO stock solutions were made and diluted with LB to obtain concentrations of 40, 20, 10, 5, 2.5, 1.25, and 0.62 mM GSNO within a 96-well plate, which also contained *E. coli* cultures (Table 1). Both the GSNO and ECΦ were plated at a 2:1 ratio with the culture volume. Once combined, the 96-well plates were immediately placed in a 37 °C Biotek Cytation Image Reader for analysis at 600 nm every 30 min over 12 h while shaking at 150 RPM. This process was done in triplicate before the results were averaged and plotted.

**Table 1:** Concentrations of GSNO and ECΦ used in Microbial Analyses and/or NO Release Tests

Solution	GSNO Conc (mM)	ECΦ Conc (PFU mL^–1^)
GNSO Only	40	-
	20	-
	10	-
	5	-
	2.5	-
	1.25	-
	0.62	-
ECΦ Only	-	10^3^
	-	10^4^
	-	10^5^
	-	10^6^
	-	10^7^
	-	10^8^
	-	10^9^
GSNO+ECΦ	10	10^6^
	5	10^6^
	2.5	10^6^

##### 2.2.5b Combined Antibacterial Analyses

ECΦ and GSNO were plated together at varying concentrations to assess their combined efficacy in bacterial killing over a 12 h period, as outlined above. However, these triplet runs combined *E. coli* cultures, ECΦ, and GSNO at a ratio of 2:1:1, respectively. Once again, a range of concentrations was tested against one another in a 12 h growth curve. Based on the results of the individual analyses, a single bacteriophage concentration was selected, along with the 3 most relevant GSNO concentrations: 2.5, 5, and 10 mM. The cultures, ECΦ, and GSNO solutions were combined in a 96-well plate and read using the BioTek Cytation Image Reader, as described above. When analyzing the OD values for each combination therapy, the corresponding controls were subtracted to ensure the decrease in OD was solely due to the presence of NO or ECΦ. These same methodologies were used to analyze the antibacterial effects of the combination solution against MRSA.

### 2.3 Statistical Analysis

All data is reported as mean ± standard deviation unless otherwise stated. Statistical analyses were run using Prism 9.2.0 (Graphpad Software, San Diego, CA). Comparisons between treatment groups and controls were determined using two-way analysis of variance (ANOVA) with corrections for multiple comparisons between means. Values of *p* < 0.05 were deemed significant; reported values of significance follow the pattern of *: *p* < 0.05, **: *p* < 0.01, and ***: *p* < 0.001.

## 3 Results

A preliminary analysis was conducted to assess the potential interactions between the two antibacterial agents before initiating any combination studies. First, a basic DLPA for *E. coli* was performed and treated with ECΦ that had been incubated for 0 and 12 h with GSNO (Figure 1a). As expected, the titer decreased slightly with longer incubation periods and higher GSNO concentrations. The growth pattern of ECΦ is illustrated in Figures 2 and 3, which helps explain the lower titers observed in Figure 1a. Nitric oxide release profiles were then analyzed using chemiluminescent nitric oxide analyzers (NOAs). Nitric Oxide Analyzers (NOAs) are the gold standard for analyzing nitric oxide release. They can detect NO levels as low as the picomolar range.^19^ These release profiles were assessed to determine the effect of bacteriophages on NO release from the endogenous donor, GSNO. The same GSNO concentrations were tested in solutions across the 12 h period with and without ECΦ. Excess GSNO in the 10 mM GSNO exhibited slightly higher NO production at the 12 h time. There was a significant decrease in the ECΦ titer after extended exposure to high levels of GSNO, but no significant decrease in NO production. Because the phage titer never dropped below the viable range of phage strength, and NO production continued after the inclusion of ECΦ, the combination was tested further.^20^

**Figure 1:**
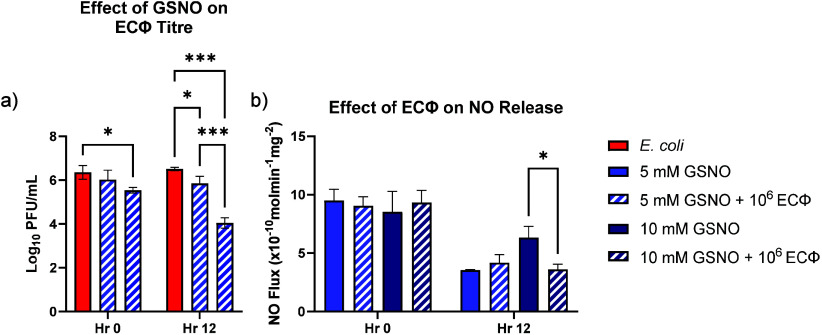
Evaluation of GSNO and ECΦ interference which one another based on bacteriophage titer (a) and NO release capabilities of GSNO in the presence of ECΦ (b). Statistical significance is represented by *: *p* < 0.05; ***: *p* < 0.001.

**Figure 2:**
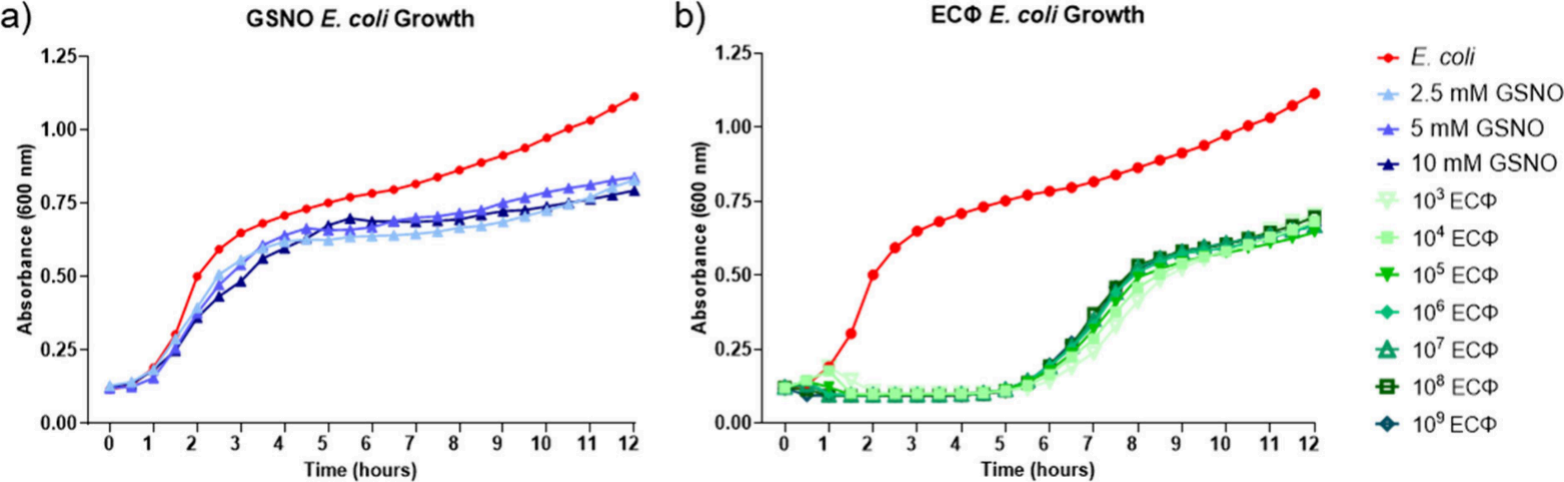
A 12-h growth analysis of *E. coli* in the presence of GSNO (a) and ECΦ (b) as antibacterial moieties.

**Figure 3:**
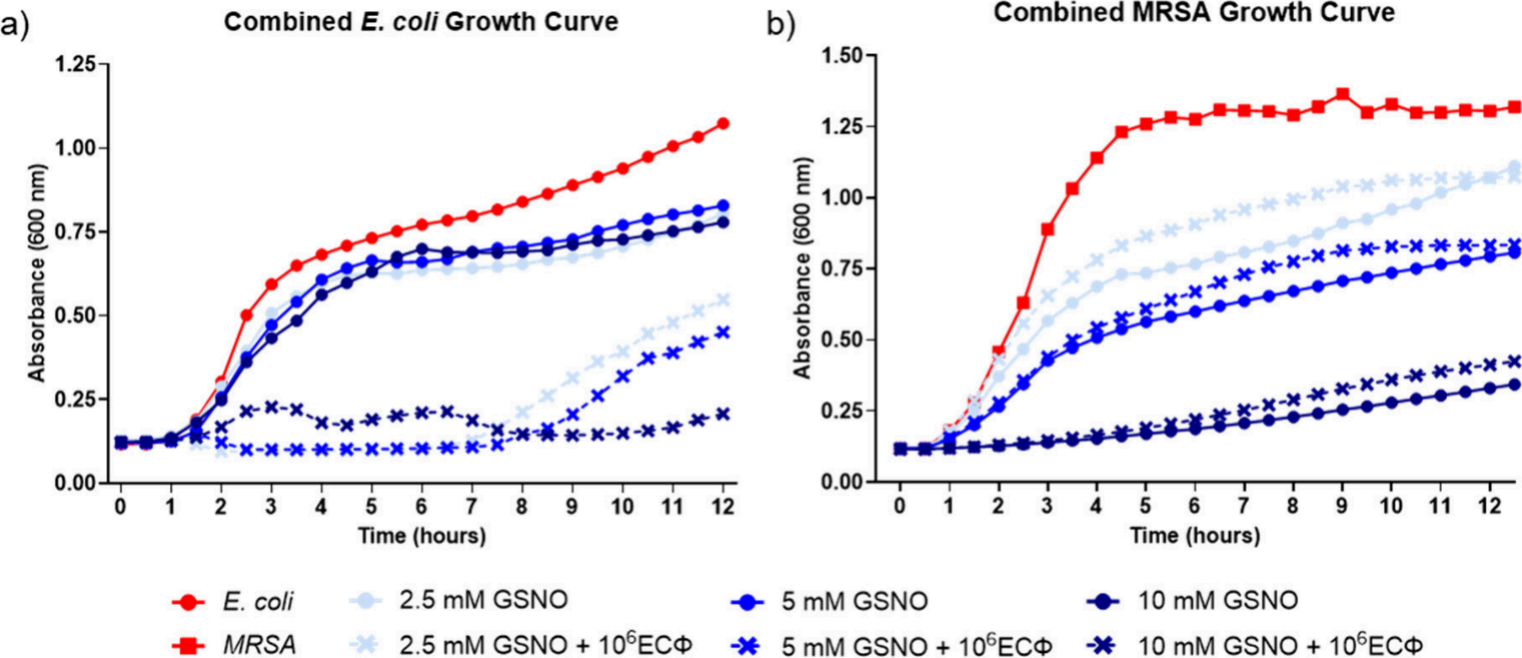
A 12 h growth curve analysis of *E. coli* (a) and MRSA (b) under combined treatment with GSNO and 10^6^ ECΦ.

Individual analyses of the bactericidal effects of GSNO and ECΦ were completed before any combined treatments were explored (Figure 2). There was a slight positive trend between inhibition level and GSNO concentration, which was not observed with the varying ECΦ titers tested. However, both therapies showed similar levels of bacterial killing after 12 h, with the OD_600_ value dropping from 1.07 ± 0.22 to 0.78 ± 0.19 and 0.67 ± 0.05 for the 10 mM GSNO concentration and all ECΦ titers, respectively, when compared to the *E. coli* control. There was also a distinct difference in growth patterns between the GSNO and the phages; NO delayed bacterial killing, as evidenced by an initial spike in OD_600_, mimicking the natural growth pattern of *E. coli* in the control suspension. GSNO acts as a prodrug and requires time to chemically decompose and release NO through S-N bond cleavage. GSNO was chosen in this study due to its controlled and delayed release of NO compared to other, more immediate, NO donors, like diazeniumdiolates. Conversely, ECΦ inhibited bacterial growth entirely for the first 6 h of the assay, after which the bacteria reemerged. In this study, no significant difference in ECΦ titers was observed after 12 h; therefore, a single titer (10^6^ PFU mL^−1^) was chosen for further experiments.

Nitric oxide-based concentration-dependent bacterial inhibition was observed with the selective ECΦ (Figures 3 and 4). The addition of ECΦ led to almost complete inhibition of bacterial growth for the first 6 h, mirroring the profile observed with ECΦ alone. Unlike ECΦ alone, the combination treatments exhibited sustained antibacterial efficacy throughout the study. The delay in *E. coli* growth significantly inhibited the overall bacterial growth when coupled with the inhibitory profile of GSNO, which was slightly opposite to ECΦ. The most significant decrease in OD_600_ after the inclusion of 10^6^ PFU mL^−1^ of ECΦ was observed with the 10 mM GSNO samples, declining from 0.78 ± 0.19 to 0.21 ± 0.03 – an 81.5 ± 1.7% decrease in bacterial growth. Lower concentrations of GSNO exhibited profiles similar to those of 10 mM GSNO but showed a less pronounced bactericidal effect when combined with ECΦ (Figure 3a). However, with the addition of ECΦ, the 5 and 2.5 mM GSNO concentrations still showed a 59.4 ± 21% and 49.9 ± 6% decrease in OD_600_ after 12 h, respectively.

**Figure 4:**
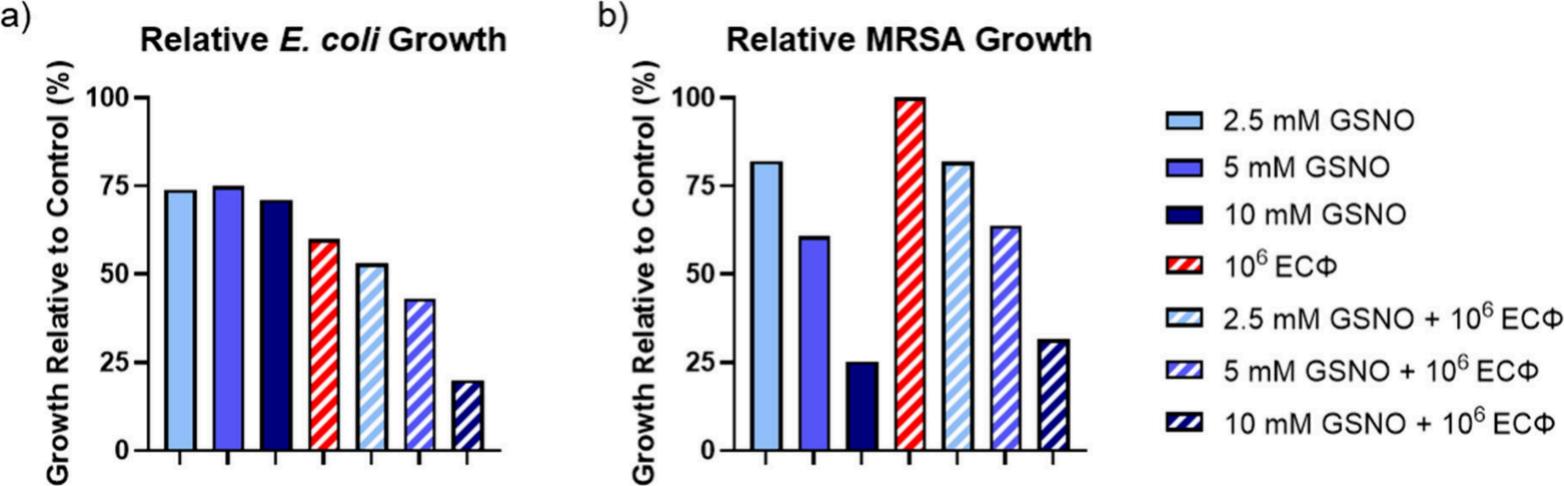
Percent bacterial growth of the combination treatments plotted with respect to control *E. coli* culture (a) or control MRSA culture (b).

With the introduction of NO as a combination solution, the broad-spectrum killing capacity of the solution was also tested. The combination treatments displayed similar dose-dependent decreases in MRSA growth compared with *E. coli*, providing 17.9 ± 5.9%, 36.0 ± 4.8%, and 68.3 ± 2.9% inhibition at 2.5, 5, and 10 mM GSNO, respectively, coupled with 10^6^ PFU mL^−1^ of ECΦ. The trends shown in Figure 3b indicate that GSNO concentrations are the most significant therapeutic factor contributing to bacterial killing of MRSA; however, the combination therapy significantly decreased bacterial growth in the phage target, *E. coli*, as well as a secondary pathogen, MRSA (Figure 4).

## 4 Discussion

To determine how bacteriophages and NO may interfere with one another’s antibacterial activity, GSNO+ECΦ were incubated together and tested to ensure their individual therapeutic potentials were not affected. Excess NO in the 10 mM GSNO samples may have led to nitrosative stress caused by reactive nitrogen species, resulting in the inactivation of the bacteriophage and bacterial host lysis.^21^ Another explanation may be the sheer number of ECΦ in the solution; it is not uncommon for phages to enter their lysogenic cycle when multiple phages attempt to enter the same host cell. A lysogenic infection does not lyse the bacterial host but allows the host to continue replicating and dividing with modified phage DNA/RNA inserted into its chromosome, generating a prophage.^22^ When placed under optimal environmental conditions, bacteria replicating in the lysogenic phase can spontaneously revert to the lytic phase. In this case, the phage titer did not fall below the effective range of titers and, therefore, was not a concern when moving forward with this combination therapy.

In the present study, the NO release characteristics of GSNO+ECΦ in a solution-based method exhibited a comparable profile to previous reports of GSNO, exhibiting increased antibacterial, antibiofilm, and antithrombotic capabilities in biomedical applications.^16^ Low levels of NO release modulation were observed during the coincubation of GSNO+ECΦ in the solution, indicating that NO was still released into the system to aid in bacterial killing. Nitric oxide was released at comparable levels across GSNO concentrations, indicating that the spike in NO release in the 10 mM GSNO samples is most likely due to autocatalysis. Autocatalysis is promoted by secondary reactions within the thiyl radicals present within primary *S*-nitrosothiols, such as GSNO, and may lead to longer-term release profiles.^23^

A notable observation was made during analysis of each therapy’s behavior when exposed to *E. coli*. Despite being both potent and well-established, the antibacterials NO and ECΦ exhibit different inhibition rates against the Gram-negative *E. coli*. Figure 2 outlines the delayed growth inhibition after exposure to GSNO coupled with the instantaneous activity of the ECΦ. In an antibacterial setting, the GSNO did not show strong bacterial growth inhibition until the 4–6 h time point, whereas the ECΦ growth peaked close to 5 h before efficacy slowly decreased at the 6–8 h time point, indicating that the bactericidal profiles for each therapy are almost exact opposites. These varying profiles have the potential to increase the antimicrobial lifespan of any future combination therapies significantly. While the GSNO requires time to break down in the system, bacteriophages can inhibit growth and increase bacteria’s susceptibility to NO. As shown, the phages decrease in efficacy several hours after introduction; the re-emergence of bacterial growth can be explained based on the nonsusceptible bacterial cells that evade phage infection during cycles of host-phage infection due to mutation of their phage-binding receptors.^24^ Although a concern in phage therapeutics, these mutations in bacterial receptors have been shown to lead to changes within the phage itself, enabling it to recognize different receptors on bacteria after multiple generations.^25,26^ These host-phage mutations have been shown to evolve rapidly, making it difficult for pathogens to develop resistance to their phage counterparts.

With the combination solution and increased concentrations of GSNO, there were substantial, more sustained bactericidal effects against *E. coli*. Similar to combination studies involving large antibiotics coupled with NO, it is anticipated that the increased bactericidal impact is due to the ability of NO to disrupt the outer bacterial membrane, leading to quicker entry for the bacteriophages present in solution.^27,28^ The phages can then replicate at a higher rate while the NO molecules continue to break down the membranes.

NO-releasing materials have shown their broad-spectrum antibacterial capabilities across numerous materials and bacterial strains based on these combined antibacterial mechanisms.^11,16^ Due to these mechanisms, the combination of NO and ECΦ showed effectiveness against other AMR pathogens, such as Methicillin resistant *S. aureus* (MRSA). In many cases, hospital-acquired infections evolve into multispecies infections; the specificity of ECΦ does not allow the broad-spectrum killing required in such scenarios. However, with the inclusion of NO in the treatment, significant reductions in viable bacterial counts were observed across multiple bacterial species. The combined therapeutics consistently showed substantial increases in bactericidal activity over the 12 h period, further supporting the potential of NO+phage biomedical therapeutics for antibacterial applications.

## 5 Conclusions

Combining two naturally occurring antibacterial molecules, NO and phages, is an idea that has yet to be explored for biomedical applications. The results herein display a promising level of combined killing compared to the GSNO and phage-only controls. With the combination of these two agents, a mechanistically complementary strategy was proven as the phages immediately invaded present bacteria while GSNO slowly broke down, gradually releasing NO. The consistent rise in NO concentration continued to prime the microenvironment, enhancing membrane permeability and resensitizing resistant cells, thereby ensuring precise and self-amplifying bacterial killing.

This preliminary study has served as the basis for developing novel biomaterials using bacteriophages coupled with NO donors in solution and in polymer matrices. This study shows compatibility and synergistic bactericidal effects between phages and the NO donor, GSNO, but fails to address more in-depth limitations of NO and phage research. Future work analyzing the combinatorial potential of NO and phages will aim to highlight these limitations. Herein, the antimicrobial potential of the combination was thoroughly explored; however, it is important to recognize potential toxicity arising from the current NO in the system, as well as the stagnation of phage activity in metabolically dormant environments. Incorporating ideas from previous works utilizing these therapeutics in biomaterials, further exploration into the immobilization and encapsulation of bacteriophages, as well as the biocompatibility of NO, can be done.^29,30^ There is significant potential to develop new biomaterials using the outlined combination; the present study seeks to provide initial confirmation that, in combination, NO and phages create an inhospitable environment for Gram-negative and Gram-positive bacteria. Investigating this dual approach against *E. coli* and MRSA addresses a unique research gap by bridging multipathogen therapeutic challenges and represents an attempt to engineer dynamic, cooperative antimicrobial systems for clinical application.

## References

[1] Golkar Z., Bagasra O., Pace D. G. (2014). Bacteriophage therapy: a potential
solution for the antibiotic resistance crisis. J Infect Dev Ctries.

[2] Boucher H. W. (2020). BAD BUGS,
NO DRUGS 2002–2020: PROGRESS, CHALLENGES, AND CALL TO ACTION. Trans Am Clin Climatol Assoc..

[3] Boucher H. W., Talbot G. H., Bradley J. S., Edwards J. E., Gilbert D., Rice L. B., Scheld M., Spellberg B., Bartlett J. (2009). Bad Bugs, No Drugs: No ESKAPE! An Update from the Infectious
Diseases Society of America. Clinical Infectious
Diseases.

[4] Wink D. A., Mitchell J. B. (1998). Chemical biology
of nitric oxide: insights into regulatory,
cytotoxic, and cytoprotective mechanisms of nitric oxide. Free Radical Biol. Med..

[5] Zaki M. H., Akuta T., Akaike T. (2005). Nitric Oxide-Induced
Nitrative Stress
Involved in Microbial Pathogenesis. J. Pharm.
Sci..

[6] Miller C., McMullin B., Ghaffari A., Stenzler A., Pick N., Roscoe D., Ghahary A., Road J., Av-Gay Y. (2009). Gaseous nitric
oxide bactericidal activity retained during intermittent high-dose
short duration exposure. Nitric Oxide.

[7] Privett B. J., Broadnax A. D., Bauman S. J., Riccio D. A., Schoenfisch M. H. (2012). Examination
of bacterial resistance to exogenous nitric oxide. Nitric Oxide.

[8] Rouillard K. R., Novak O. P., Pistiolis A. M., Yang L., Ahonen M. J. R., McDonald R. A., Schoenfisch M. H. (2021). Exogenous
Nitric Oxide Improves Antibiotic
Susceptibility in Resistant Bacteria. ACS Infect.
Dis..

[9] Devine R., Douglass M., Ashcraft M., Tayag N., Handa H. (2021). Development
of Novel Amphotericin B-Immobilized Nitric Oxide-Releasing Platform
for the Prevention of Broad-Spectrum Infections and Thrombosis. ACS Applied Materials &amp; Interfaces.

[10] Mondal A., Singha P., Douglass M., Estes L., Garren M., Griffin L., Kumar A., Handa H. (2021). A Synergistic New Approach
Toward Enhanced Antibacterial Efficacy via Antimicrobial Peptide Immobilization
on a Nitric Oxide-Releasing Surface. ACS Appl.
Mater. Inter..

[11] Estes
Bright L. M., Garren M. R. S., Douglass M., Handa H. (2023). Synthesis
and Characterization of Nitric Oxide-Releasing Ampicillin as a Potential
Strategy for Combatting Bacterial Biofilm Formation. ACS Applied Materials &amp; Interfaces.

[12] Bacteriophages: Methods and Protocols; Martha, R. J. C. , Andrew, K. , Lavigne, R. , Eds.; Vol. 4; Springer Science+Business Media, LLC, 2019.

[13] Ho K. (2001). Bacteriophage
Therapy for Bacterial Infections: Rekindling a Memory from the Pre-Antibiotics
Era. Perspect. Biol. Med..

[14] Kutateladze M., Adamia R. (2010). Bacteriophages as potential
new therapeutics to replace
or supplement antibiotics. Trends in Biotechnology.

[15] Matsuzaki S., Rashel M., Uchiyama J., Sakurai S., Ujihara T., Kuroda M., Ikeuchi M., Tani T., Fujieda M., Wakiguchi H. (2005). Bacteriophage therapy: a revitalized therapy
against bacterial infectious diseases. J Infect
Chemother.

[16] Ashcraft M., Douglass M., Garren M., Mondal A., Bright L. E., Wu Y., Handa H. (2022). Nitric Oxide-Releasing Lock Solution for the Prevention
of Catheter-Related Infection and Thrombosis. ACS Appl. Bio. Mater..

[17] Gondil V. S., Ashcraft M., Ghalei S., Kumar A., Wilson S. N., Devine R., Handa H., Brisbois E. J. (2025). Anti-infective bacteriophage
immobilized nitric oxide-releasing biomaterial for prevention of thrombosis
and device associated infections. ACS Appl Bio
Mater.

[18] Ackermann, H.-W. Phage Classification and Characterization. In Bacteriophages: Methods and Protocols, Volume 1: Isolation, Characterization, and Interactions, Clokie, M. R. J. , Kropinski, A. M. Eds.; Humana Press, 2009; pp 127-140.

[19] Maniscalco M., Vitale C., Vatrella A., Molino A., Bianco A., Mazzarella G. (2016). Fractional
exhaled nitric oxide-measuring devices:
technology update. Med. Devices: Evid. Res..

[20] Abedon, S. Chapter 1 - Phage Therapy Pharmacology: Calculating Phage Dosing. In Adv. Appl. Microbiol., Laskin, A. I. , Sariaslani, S. , Gadd, G. M. Eds.; Vol. 77; Academic Press, 2011; pp 1-40. 10.1016/B978-0-12-387044-5.00001-7 22050820

[21] Barraud N. (2006). Involvement of Nitric Oxide in Biofilm Dispersal of Pseudomonas aeruginosa. J. Bacteriol..

[22] Marchi J., Zborowsky S., Debarbieux L., Weitz J. S. (2023). The dynamic interplay
of bacteriophage, bacteria and the mammalian host during phage therapy. iScience.

[23] De
Oliveira M. G., Shishido S. M., Seabra A. B., Morgon N. H. (2002). Thermal
Stability of Primary < i > S</i>-Nitrosothiols: Roles
of Autocatalysis
and Structural Effects on the Rate of Nitric Oxide Release. J Physic. Chem. A.

[24] Silva-Valenzuela C. A., Camilli A. (2019). Niche adaptation limits
bacteriophage predation of
< i > Vibrio cholerae</i> in a nutrient-poor aquatic environment. Proc. Natl. Acad. Sci.

[25] Drexler K., Dannull J., Hindennach I., Mutschler B., Henning U. (1991). Single mutations in a gene for a
tail fiber component
of an Escherichia coli phage can cause an extension from a protein
to a carbohydrate as a receptor. J. Mol. Biol..

[26] Hampton H. G., Watson B. N. J., Fineran P. C. (2020). The arms race between bacteria and
their phage foes. Nature.

[27] Ren H., Wu J., Colletta A., Meyerhoff M. E., Xi C. (2016). Efficient Eradication
of Mature Pseudomonas aeruginosa Biofilm via Controlled Delivery of
Nitric Oxide Combined with Antimicrobial Peptide and Antibiotics. Front. Microbiol..

[28] Wu Y., Garren M. R., Estes-Bright L. M., Maffe P., Brooks M., Brisbois E. J., Handa H. (2024). Enhanced antibacterial efficacy against
antibiotic-resistant bacteria via nitric oxide-releasing ampicillin
polymer substrates. J. Colloid Interface Sci..

[29] Hopkins S. P. (2018). Achieving Long-Term
Biocompatible Silicone via Covalently Immobilized
S-Nitroso-N-acetylpenicillamine (SNAP) That Exhibits 4 Months of Sustained
Nitric Oxide Release. ACS Appl. Mater. Interfaces.

[30] Brisbois E. J., Handa H., Major T. C., Bartlett R. H., Meyerhoff M. E. (2013). Long-term
nitric oxide release and elevated temperature stability with S-nitroso-N-acetylpenicillamine
(SNAP)-doped Elast-eon E2As polymer. Biomater.
Sci..

